# Musculoskeletal manifestations in Alkaptonuria: A cross-sectional study

**DOI:** 10.1097/MD.0000000000028241

**Published:** 2021-12-23

**Authors:** Aysha Habib Khan, Bushra Afroze, Hafsa Majid, Yusra Zaidi, Azeema Jamil, Lena Jafri

**Affiliations:** a Department of Pathology& Laboratory Medicine, Faculty of Pathology, Aga Khan University, Karachi, Pakistan; b Department of Pediatrics and Child Health, Faculty of Pediatrics, Aga Khan University, Karachi, Pakistan.

**Keywords:** alkaptonuria, homogentisic acid, musculoskeletal, Pakistan

## Abstract

This study aimed to determine the patient characteristics and clinical presentation of Alkaptonuria cases reported by the Biochemical Genetics Lab.

An observational study was conducted at the Biochemical Genetics Lab. Alkaptonuria patients were diagnosed based on the homogentisic acid peak in urine and their demographics and clinical data collected from to 2013 to 2019. Clinical history related to joint diseases, ochronotic presentation, and urine darkening on standing was collected.

During 7 years, 21 Alkaptonuria cases were reported from BGL; mean age 19.4 ± 24.5 years (range 0.2–66 years) and male to female ratio of 2:1. Of the total, only 9 were adults (mean age, 44 ± 12 years). Most adult patients had musculoskeletal involvement, with joint pain (n = 9) and ochronotic pigmentation (n = 6), whereas all patients presented with a history of urine darkening on standing (21/21 cases).

The high prevalence of musculoskeletal involvement observed in patients with albuminuria is likely to be missed by physicians unless specifically tested for in such cases.

## Abstract 2

Supplemental Digital Content is available in the text

## 1 Introduction

Alkaptonuria is a rare autosomal recessive metabolic disorder caused by absolute or relative deficiency of the hepatic enzyme homogentisate 1, 2-dioxygenase (HGO).^[1]^ The true incidence of alkaptonuria is unknown, and there is wide variability in the reported incidences. For example, in the National Registry of the UK, the incidence of albuminuria is 1 in 500,000, whereas it is 25 in 500,000 in Slovakia, the Dominican Republic, and the Middle East. This high incidence in Slovakia, the Dominican Republic, and the Middle East could be due to the high rate of intermarriages in these populations.^[2–5]^

**Figure 1:**
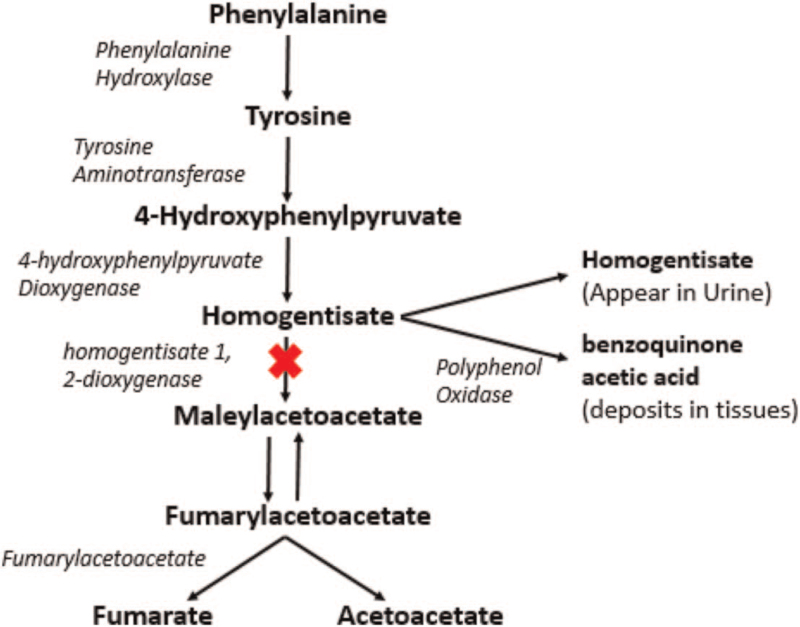
The metabolic pathway of tyrosine metabolism, showing block in the pathway at the level of homogentisate 1, 2-dioxygenase leading to Alkaptonuria.

The enzyme HGO forms an intermediate metabolite, homogentisic acid (HGA), in the tyrosine degradation pathway, which appears in urine when HGO is deficient, as shown in Figure 1.^[6]^ The HGA is oxidized to black-colored benzoquinone acetic acid, which causes the urine of patients to turn black on standing and is a pathognomonic finding in patients with albuminuria.^[7]^ Benzoquinone acetic acid is converted into a melanin-like pigment, which polymerizes with connective tissue, discoloring the tissues, and making them brittle, weak, and susceptible to rupture.^[8,9]^ This pigment deposition leads to poor quality of life and significant alkaptonuria-induced multimorbidity.^[10]^

The present study aimed to determine the patient characteristics and clinical presentation of Alkaptonuria cases reported by the Biochemical Genetics Lab (BGL).

## 2 Materials and methods

An observational study was conducted at BGL in the Department of Chemical Pathology, Department of Pathology and Laboratory Medicine, Aga Khan University, Karachi, Pakistan. Approval from the institute's Ethical Review Committee (ERC) was obtained (5085-Pat-ERC-17) before the commencement of the study. All patients tested for urinary organic acid (UOA) analysis from January 2013 to December 2019 were reviewed, and only those who presented with alkaptonria, including those diagnosed with other inherited metabolic disorders, were excluded. Clinical details were collected by telephone interviews with patients or guardians after verbal informed consent. Details including demographics, birth, drug and family history, symptomatology, and clinical manifestations related to joint involvement were captured on a structured clinical history form.

**Figure 2:**
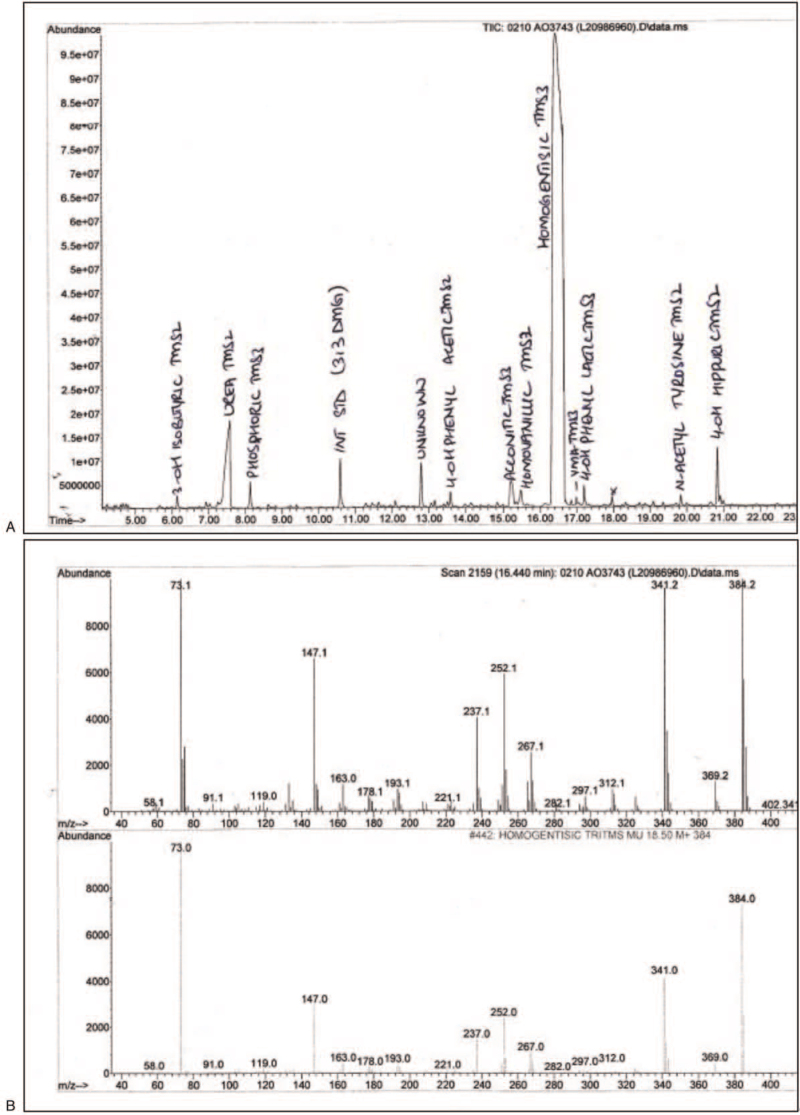
(A) Urine organic acid chromatogram performed by gas chromatography-mass spectrometry, showing a peak of homogentisic acid (HGA). The *x*-axis represents the retention/elution time, while the *y*-axis represents the abundance of molecules. HGA appears as a trimethylsilyl derivative at a retention time of 16 minutes, measured against an internal standard (3,3 dimethylglutarate). HGA is reported as a large, moderate, or small peak relative to the peak of the internal standard. (B) Qualifying ion spectrum of homogentisic acid. The *x*-axis shows the mass to charge ratio, while on the *y*-axis is the abundance of molecules, the above picture shows the qualifying ion spectrum of an Alkaptonuria patient compared to the mass to charge ratio of homogentisic acid from qualifying ions reference library shown in the lower figure. The qualifying ions of homogentisic acid were 341, 284, and 252.

Urine organic acid was analyzed by gas chromatography-mass spectrometry (GCMS) on an Agilent GC-MS system (Agilent Technologies, US) using ethyl acetate and derivatization by bis (trimethylsilyl) acetamide compound. The 3, 3 Dimethyl glutaric acid is used as an internal standard. Patients were diagnosed with Alkaptonuria based on the presence of an HGA peak on the UOA chromatogram, appearing at a retention time of 16 minutes (Fig. 2A). Ion spectrum matching of HGA was performed with the qualifying ion reference library (Fig. 2B).

## 3 Results

Twenty-one cases of alkaptonuria were reported over a 7-year period. The mean and standard deviation of the patients was mean age 19.4 ± 24.5 years (range 0.2–66 years) and male to female ratio of 2:1 (Table 1). Nine patients presented after the second decade of life, whereas 8 cases presented within the infantile period. Parental consanguinity was present in 15 of 21 patients with alkaptonuria. The pathognomonic feature of urine darkening on standing or dark-colored staining of underclothing was observed in all patients (21/21). Most patients had symptoms for 12.2 ± 6.6 years (range 3–25 years).

**Table 1:** Clinical presentation of patients with alkaptonuria reported by BGL.

						Common clinical features		
No.	Year of UOA testing/publication	City of residence	Sex	Presenting age in years	Duration of symptoms in years	Ochronotic pigmentations	Joint pain	Urine darkening on standing
Biochemical genetics laboratory data, n = 12 (2013–2019)								
1.	2019	Nawab Shah	M	66	25	Yes	Yes	Yes
2.	2019	Rawalkot	F	47	15	Yes	Yes	Yes
3.	2019	Karachi	F	0.8	–	No	No	Yes
4.	2019	Peshawar	M	1	–	No	No	Yes
5.	2019	Rawalkot	M	36	7	No	Yes	Yes
6.	2018	Lahore	M	0.5	–	No	No	Yes
7.	2018	Hyderabad	M	2.2	–	No	No	Yes
8.	2018	Karachi	M	0.2	–	No	No	Yes
9.	2018	Karachi	F	1.5	–	No	No	Yes
10.	2018	Karachi	M	1.7	–	No	No	Yes
11.	2017	Lahore	M	28	10	No	Yes	Yes
12.	2017	Rawalpindi	F	35	8	Yes	Yes	Yes
13.	2017	Karachi	M	1.8	–	No	No	Yes
14.	2017	Karachi	F	0.75	–	No	No	Yes
15.	2016	Karachi	F	0.9	–	No	No	Yes
16.	2016	Lahore	M	55	20	Yes	Yes	Yes
17.	2016	Hyderabad	M	0.1	–	No	No	Yes
18.	2016	Lahore	M	57	15	Yes	Yes	Yes
19.	2015	Lahore	M	0.3	–	No	No	Yes
20.	2015	Lahore	M	40	7	No	Yes	Yes
21.	2014	Rawalpindi	F	33	3	Yes	Yes	Yes

F = female, M = male, UOA = urine organic acid.

Musculoskeletal involvement, low back pain, and stiffness followed by chronic knee joint pain were observed in adult patients only; joint pain in 9 of 21 patients (Supplemental figure 1, http://links.lww.com/MD/G553 shows the knee radiographs of an AKU patient). Ochronotic pigmentation on the face/hands and ear cartilage was seen in 4 and 2 patients, respectively. Apart from 1 patient with mixed renal stones, none of the patients developed any cardiac or renal complications associated with albuminuria. All patients were symptomatically treated with ascorbic acid, NSAIDS, prescribed a low-protein diet, and physiotherapy, but none were on Nitosone therapy.

## 4 Discussion

Alkaptonuria, a rare disease caused by subtle signs and symptoms, is frequently under-diagnosed. Only 12 cases have been reported from Pakistan until 2019, with the first report published in 2005. Patients are often evaluated for porphyria, which also has the same feature of darkening of urine while standing as Alkaptonuria. These patients are often diagnosed late, which leads to poor quality of life. In infants, the staining of diapers is an essential early diagnostic clue for albuminuria, which parents and physicians often miss. In addition, fewer laboratories are performing sensitive and specific tests for the diagnosis of alkaptonuria, which have led to the loss of patients. Moreover, this became evident after the availability of the right diagnostic tool (UOA evaluation by GCMS), 21 cases were diagnosed in only 7 years, whereas literature reports only 12 cases from Pakistan in >70 years.

## 5 Characteristics of Alkaptonuria patients reported from Pakistan

A review of published literature from 1947 to 2019 identified 9 case reports and 1 original study reporting alkaptonuria in 3 patients from Pakistan (Table 2). In all the reports except one, the presence of HGA in urine was confirmed by colorimetry; 1 study utilized paper chromatography to identify HGA, whereas none utilized GCMS for UOA analysis.^[6–8,11–13]^ The medical specialties where patients presented initially included medicine (n = 3), rehabilitation medicine (n = 2), dermatology (n = 3), laboratory (n = 3), and radiology (n = 1). A male predominance was observed in these case reports. Only 1 report mentioned the consanguinity of the patient's parents. The most common age of presentation was in the fourth or fifth decade of life, with 1 case presented in their 20s and infancy. Clinical details were available for only 9 patients. Adult patients had symptoms for 10 to 15 years. A family history of alkaptonuria was provided in 4 of 9 case reports, and 1 to 2 siblings of each patient were affected. Musculoskeletal involvement was seen in all cases from the literature; all cases had joint pain, 5 of 9 also reported restriction movements, whereas 1 patient each had a height loss of 15 cm and flexion contractures. Except for 1 patient who developed renal stones, none had cardiac or renal complications. Arthritis, notably in the spine and large joints (Ochronotic musculoskeletal manifestations), was a common presentation observed in both patients reported from BGL and in a literature review from Pakistan.

**Table 2:** Review of case reports published from Pakistan on Alkaptonuria (1996–2016).

S#	Publication year	Specialty of presentation	Sex	Age at presenting. y	Duration of symptoms	Family history of AKU	Clinical presentation
1	1996	Medicine (Peshawar)	M	45	11	A brother and niece have AKU	Arthritis (wrists, elbows, shoulders, knees, ankles) Restricted spinal movements. Ochronotic pigmentation of ear cartilage Brownish discoloration of sclera Renal stones which turn black on exposure to air Staining of vests and clothes by his sweat.
2	2008	Pediatrics (Lahore)	F	1 month	Not recorded	Elder sister (6 y) has AKU	Urine darkens on standing
3	2009	Dermatology (Karachi)	M	48	10	Not asked	Recurrent joint pains. Swollen and tenderleft knee joint. Difficulty walking. **Ochronotic pigmentation of hands.** Bluish yellow hyperkeratotic papules and plaques along the margins of palms, sides of fingers and thumbs suggestive of Keratoelastoidosis marginalis. Similar pigmentation was also prominent on his cheeks, forehead, ear lobes and axillae. Nails were markedly discolored. Eyes showed dark brown to black pigment deposition
4	2010	Radiology/pathology )Lahore)	M	38	2	Not present in family	Pain in joints (knees, shoulders, elbows, lower back) Progressive stiffness & restricted movement of spine Ochronotic pigmentation of ear cartilage
5	2005	Dermatology (Quetta)	M	26	3	Not present in family	Pain in joints (lower back, knees) **Ochronotic pigmentation of ear cartilage** Urine darkens on standing Black cerumen in ears
6	2013	Medicine (Azad Kashmir)	M	55	15	Brother has it	Pain in joints (lower back) Difficulty in walking, uses 2 sticks to support himself. Presence of kyphosis. Over the years, Macular and Hyperkeratotic **papular ochronotic discolouraton of skin over face and hands.** Osler spots on sclerae.
7	2016	Rehabilitation medicine (Lahore)	M	48	15	Father has it	Chronic pain in lower back and both knees. Loss of height from 165 cm to 150 cm. **Ochronotic papules on bilateral extensor surface of second finger.** Gray pigmentation of both sclerae.
8	2016	Rehabilitation medicine (Lahore)	F	60	20	Not present in family	Chronic aching pain in both knees and dorsal spine. Flexion contractures of both knees. Bilaterally knee crepitus present. **Ochronotic patches over her nose, proximal palms, index fingers, thumbs, dorsal foot.** Macules in sclera and over face, ears, and knees.

Long-standing arthritis in patients with albuminuria can be severe and disabling.^[14,15]^ Typically, OCM begins in the third decade of life, which is earlier than autoimmune musculoskeletal manifestations and progresses more rapidly in males than in females.^[13,14]^ Ochronotic pigmentation was observed in the ear cartilage and hands/face in 2 and 6 cases, respectively. Ochronotic pigmentation was frequently reported in the literature, whereas in the BGL data, only 6 of 21 patients had it. Clinical data obtained from BGL also identified arthritis as the most common presentation in patients. Most of these patients had symptoms for 3 to 25 years before being diagnosed with albuminuria, which may be due to the slow progression and limited awareness regarding this disease. Physicians involved in managing arthritis must specifically consider albuminuria in their differentials and inquire about other symptoms, notably the darkening of urine while standing.^[15]^

All patients with kaptonuria reported from the BGL and in the published literature were symptomatically treated and were mainly on analgesics. Few of the patients were on vitamin C supplementation; however, none were on dietary restriction, low-protein diet, physiotherapy, or nitisinone therapy. Few patients did not know which specialist to contact and had seen several general practitioners for their complaints.

The standard treatment for alkaptonuria aims to target specific symptoms of each individual. Pain management is tailored according to a person's symptoms, and narcotics may also be prescribed if needed. Dietary protein restrictions are usually ineffective in patients with albuminuria and can lead to further complications.^[10]^ Vitamin C supplementation is advised, as it prevents the accumulation and deposition of HGA. However, studies are lacking regarding its effectiveness in long-term usage. Nitisinone, an FDA-approved drug for tyrosinemia, is presently under clinical trials for its potential use in Alkaptonuria.^[16]^ Research has shown that it reduces HGA formation by diverting the metabolism toward the formation of 4-hydroxyphenyl lactic acid.^[17]^ There is a limited global experience of long-term outcomes of nitisinone treatment and its effectiveness in young children. More research is needed to evaluate its effect on preventing the deposition of quinone derivatives of HGA in connective tissues and its safety or effectiveness in long-term usage in all age groups. Approximately half of the patients with albuminuria require surgery of their hip, knee, or shoulder joints, usually by the fifth or sixth decade of life.^[18]^ Physiotherapy and occupational therapy are also advised to patients, as they help improve joint mobility and maintain the strength and flexibility of muscles and joints.^[19]^

To the best of our knowledge, this is the first national-level data from Pakistan reporting the clinical spectrum of albuminuria in our population. However, this study has several limitations, including a cross-sectional study design, a small number of Alkaptonuria cases, and the nonavailability of a confirmatory enzyme or mutational analysis. In addition, detailed clinical examination of the joints could not be performed in patients to determine the cause of musculoskeletal manifestations.

## 6 Conclusion

The present study suggests that the burden of Alkaptonuria goes unnoticed and not documented until chronic symptoms and signs of HGA deposition in tissue develop. The prevalence of albuminuria in Pakistan may be higher than that reported in the literature. Alkaptonuria is a rare autosomal recessive disorder of metabolism that is likely to be missed by physicians as a cause of musculoskeletal manifestations unless individually examined in such cases. There is a need to raise awareness among health practitioners to look for albuminuria in unexplained musculoskeletal manifestations.

## Author contributions

AHK was included in conceiving ideas, reviewing chromatograms, and critically reviewing the manuscript for intellectual content. BA reviewed the manuscript for intellectual content. HM was involved in the data analysis and writing manuscripts. YZ was involved in data collection, literature search, and manuscript writing. AJ was involved in the UOA analysis and chromatogram review. LJ was included in reviewing chromatograms, literature searches, and critically reviewed the manuscript for intellectual content.

**Conceptualization:** Aysha Habib Khan.

**Data curation:** Hafsa Majid, Yusra Zaidi.

**Formal analysis:** Hafsa Majid.

**Investigation:** Azeema Jamil.

**Methodology:** Yusra Zaidi.

**Project administration:** Hafsa Majid.

**Supervision:** Aysha Habib Khan.

**Validation:** Bushra Afroze, Hafsa Majid, Lena Jafri.

**Writing – original draft:** Hafsa Majid.

**Writing – review & editing:** Aysha Habib Khan, Bushra Afroze, Yusra Zaidi, Azeema Jamil, Lena Jafri.

## References

[1] Mistry JB Bukhari M Taylor AM. Alkaptonuria. Rare Dis 2013;1:e27475. 25003018 10.4161/rdis.27475 PMC3978898

[2] Zatkova A de Bernabe DB Polakova H. High frequency of Alkaptonuria in Slovakia: evidence for the appearance of multiple mutations in HGO involving different mutational hot spots. Am J Hum Genet 2000;67:1333–9. 11017803 10.1016/s0002-9297(07)62964-4 PMC1288576

[3] Milch RA. Studies of Alcaptonuria: inheritance of 47 cases in eight highly inter-related Dominican kindreds. Am J Hum Genet 1960;12:76–85. 17948450 PMC1932065

[4] Al-Sbou M Mwafi N. Nine cases of Alkaptonuria in one family in southern Jordan. Rheumatol Int 2012;32:621–5. 21127875 10.1007/s00296-010-1701-1

[5] Phornphutkul C Introne WJ Perry MB. Natural history of Alkaptonuria. N Engl J Med 2002;347:2111–21. 12501223 10.1056/NEJMoa021736

[6] Din RU Rizvi SD Naqeeb M Masud S. Alkaptonuria and keratoelastoidosis marginal an unusual association. PAFMJ 2009;59:240–2.

[7] Nafees M Muazzam M. Alkaptonuria-case report and review of literature. Pak J Med Sci 2007;23:650.

[8] Ranjha KM Aslam S Mahmood T. Alkaptonuria-a rare metabolic disorder. J Pak Assoc Dermatol 2005;15:351–4.

[9] Taylor AM Vercruysse KP. Analysis of melanin-like pigment synthesized from homogentisic acid, with or without tyrosine, and its implications in Alkaptonuria. JIMD Rep 2017;35:79–85. 27943071 10.1007/8904_2016_27 PMC5585104

[10] Ranganath LR Jarvis JC Gallagher JA. Recent advances in management of Alkaptonuria (invited review; best practice article). J Clin Pathol 2012;66:367–73. 10.1136/jclinpath-2012-200877 23486607

[11] Ahmed S Shah Z Ali N. Chronic low backache and stiffness may not be due ankylosing spondylitis. J Pak Med Assoc 2010;60:681–3. 20726204

[12] Alam I Ahmed H Bangash M Khan N Biland B. Case report of alkaptonuria. J Postgrad Med Inst (Peshawar-Pakistan) 2011;10:

[13] Rathore FA Ayaz SB Mansoor SN. Ochronotic arthropathy: two case reports from a developing country. Clinical Med Insights Arthritis Musculoskelet 2016;9: CMAMD. S31560. 10.4137/CMAMD.S31560 PMC4749042 26884684

[14] Saigal R Tank M Pathak P Choudhary A Saigal S. Alkaptonuric ochronosis. J Assoc Physicians India 2016;64:79. 27734648

[15] Kanniyan K Pathak AC Dhammi IK Jain AK. Does your patient's urine turns dark? Alkaptonuria and low back ache: a literature review. J Orthop Case Rep 2014;4:29. 10.13107/jocr.2250-0685.220 PMC4719265 27298997

[16] Davison AS Norman B Milan AM. Assessment of the effect of once daily nitisinone therapy on 24-h urinary metadrenalines and 5-hydroxyindole acetic acid excretion in patients with alkaptonuria after 4 weeks of treatment. JIMD Rep 2018;41:01–10. 10.1007/8904_2017_72 PMC6122050 29147990

[17] Milan AM Hughes AT Davison AS. The effect of nitisinone on homogentisic acid and tyrosine: a two-year survey of patients attending the National Alkaptonuria Centre, Liverpool. Ann Clin Biochem 2017;54:323–30. 28081634 10.1177/0004563217691065

[18] Karaoğlu S Karaaslan F Mermerkaya MU. Long-term result of arthroplasty in the treatment of a case of ochronotic arthropathy. Acta Orthop Traumatol Turc 2016;50:584–6. 27817975 10.1016/j.aott.2016.08.018 PMC6197303

[19] Arnoux JB Le Quan Sang KH Brassier A. Old treatments for new insights and strategies: proposed management in adults and children with Alkaptonuria. J Inherit Metab Dis 2015;38:791–6. 25860819 10.1007/s10545-015-9844-6

